# Remote sensing monitoring of land restoration interventions in semi-arid environments with a before–after control-impact statistical design

**DOI:** 10.1016/j.jag.2017.02.016

**Published:** 2017-07

**Authors:** Michele Meroni, Anne Schucknecht, Dominique Fasbender, Felix Rembold, Francesco Fava, Margaux Mauclaire, Deborah Goffner, Luisa M. Di Lucchio, Ugo Leonardi

**Affiliations:** a European Commission, Joint Research Centre, Directorate D – Sustainable Resources, Food Security Unit, Via Fermi 2749, 21027 Ispra, VA, Italy; b International Livestock Research Institute, P.O. Box 30709, 00100 Nairobi, Kenya; c University of Bordeaux 3, Labex DRIIHM and Les Afriques dans le monde (LAM), IEP de Bordeaux, allée Ausone 11, Domaine universitaire, 33607 Pessac Cedex, France; d French National Centre for Scientific Research, CNRS/UMI n° 3189 – Environment, Health and Societies, Bd Pierre Dramard 51, 13344 Marseille Cedex 15, France; e University of Copenhagen, Department of Geosciences and Natural Resource Management, Rolighedsvej 23, 1958 Frederiksberg, Denmark; f Food and Agriculture Organization of the United Nations, Somalia Water and Land Information Management Project, P. O. Box 30470-00100, Nairobi, Kenya

**Keywords:** Restoration interventions, Biophysical impact, Landsat, MODIS, BACI sampling design

## Abstract

Restoration interventions to combat land degradation are carried out in arid and semi-arid areas to improve vegetation cover and land productivity. Evaluating the success of an intervention over time is challenging due to various constraints (e.g. difficult-to-access areas, lack of long-term records) and the lack of standardised and affordable methodologies. We propose a semi-automatic methodology that uses remote sensing data to provide a rapid, standardised and objective assessment of the biophysical impact, in terms of vegetation cover, of restoration interventions. The Normalised Difference Vegetation Index (NDVI) is used as a proxy for vegetation cover. Recognising that changes in vegetation cover are naturally due to environmental factors such as seasonality and inter-annual climate variability, conclusions about the success of the intervention cannot be drawn by focussing on the intervention area only. We therefore use a comparative method that analyses the temporal variations (before and after the intervention) of the NDVI of the intervention area with respect to multiple control sites that are automatically and randomly selected from a set of candidates that are similar to the intervention area. Similarity is defined in terms of class composition as derived from an ISODATA classification of the imagery before the intervention. The method provides an estimate of the magnitude and significance of the difference in greenness change between the intervention area and control areas. As a case study, the methodology is applied to 15 restoration interventions carried out in Senegal. The impact of the interventions is analysed using 250-m MODIS and 30-m Landsat data. Results show that a significant improvement in vegetation cover was detectable only in one third of the analysed interventions, which is consistent with independent qualitative assessments based on field observations and visual analysis of high resolution imagery. Rural development agencies may potentially use the proposed method for a first screening of restoration interventions.

## Highlights

• A rapid, standardised and objective assessment of the biophysical impact of restoration interventions is proposed.
• The intervention impact is evaluated by a before–after control-impact sampling design.
• The method provides a statistical test of the no-change hypothesis and the estimation of the relative magnitude of the change.
• The method is applicable to NDVI and other remote sensing-derived variables.

## 1 Introduction

Desertification, defined as land degradation in arid, semi-arid and dry sub-humid areas resulting from various factors, including climate variation and human activities (UNCCD, 1994), represents a major threat to populations and ecosystems (Low, 2013, Reynolds et al., 2007). Besides physically affecting ecosystems, land degradation causes various socio-economic problems, such as food insecurity and conflicts (Mbow et al., 2015). Restoration interventions are among the strategies that can be put in place to combat land degradation. Restoration actions often involve the improvement of vegetation cover (Zucca et al., 2015), through the planting of appropriate species (e.g. Niang et al., 2014) or through improved soil, water and land management.

The definition of “effectiveness” of a restoration action may cover different aspects of the intervention, ranging from the purely biophysical to the ecological and socio-economic ones (Shackelford et al., 2013). With respect to the biophysical impact, guidelines for the ecological evaluation of restoration interventions focus on the comparison between the restoration and reference sites for a number of attributes measured in the field, ranging from species composition, to ecosystem function and stability, and to landscape context (Society for Ecological Restoration International Science & Policy Working Group, 2004). Although comprehensive, this approach is expensive and requires extensive field operations.

Independent assessment of the success of restoration projects is often challenging because interventions may be located in areas that are difficult to access and have poor infrastructure. Additional challenges refer to the lack of affordable and standardised methodologies/criteria and the difficulty of obtaining long-term data to monitor the effect of an intervention outside the project's timespan. Verification performed by the implementing agent is also frequently not available. For example, in a recent survey of restoration projects in the Mediterranean Basin conducted by Nunes et al. (2016) among restoration professional practitioners, restoration success was not evaluated in 22% of the projects and evaluated only in the first year after the plantation in 19% of the projects. When conducted, the evaluation was based on plant cover and diversity (69% of the projects) and plant vitality (48%). Lack of funds, together with capacity constraints and lack of knowledge, were identified as obstacles to project monitoring by restoration practitioners in South Africa (Ntshotsho et al., 2015) and can be assumed to represent common limitations in other rural areas across the continent.

The lack of evaluation and dissemination of the results of restoration still represent a constrain on the application of the best technologies and approaches available (Bautista et al., 2010). As a results, there is widespread consensus on the need for innovative approaches for the systematic evaluation of the effectiveness of restoration actions (Bautista et al., 2010, Benayas et al., 2009, Birch et al., 2010, Papanastasis et al., 2015).

Remote sensing (RS) can help cope with the widespread lack of timely, long-term, reliable, and homogeneous ground information, especially in African drylands. Few examples of the use of RS data to assess restoration interventions are available. The Food and Agriculture Organisation – Somalia Water and Land Information Management project (FAO-SWALIM) uses commercial very high resolution (VHR) imagery to visually appraise the implementation of surface run-off control infrastructures in Somalia (e.g. rock dams, gabions, water catchments) operated by various contactors (FAO, 2015). In this way, however, it is the implementation of the infrastructure that is scrutinised, not its impact or success with respect to vegetation dynamics. Photointerpretation of time series of aerial photography was used by Rango et al. (2002) to qualitatively evaluate the long-term effectiveness of restoration interventions in New Mexico in terms of persistency in time of recognisable structures such as terraces, grubbing patterns, revegetated areas, etc. Recently, the Openforis initiative of the FAO provided a free and open-source tool, named Collect Earth, which facilitates the visual interpretation of VHR time series imagery of Google Earth and Microsoft Bing for point sampling and land use change detection (Bey et al., 2016). Despite their usefulness, the results of the analysis are prone to interpretation errors as all of these examples make use of photointerpretation. A quantitative evaluation of a restoration intervention using *Atriplex nummularia* plantations in Morocco was instead performed by Zucca et al. (2015) utilising SPOT5 imagery and ground-based biomass measurements to derive the dry biomass yield of the plantations in Morocco as compared to known references. Land cover classification and spatial pattern metrics have been analysed by Fava et al. (2015) to study the impact of restoration actions in Mediterranean rangelands.

Vegetation indices such as the Normalised Difference Vegetation Index (NDVI; Rouse et al., 1974) can be used as proxies to monitor the fraction of vegetation cover, i.e. the fraction of ground covered by green vegetation (Carlson and Ripley, 1997). However, evaluating the “greening” of a restoration intervention presents a challenge, because the direct comparison of the NDVI of the area before and after the intervention would not be informative. In fact, vegetation cover will change over time independently of the restoration project. Two main sources drive the temporal variability of vegetation status: the annual seasonal development cycle (one or more) and the inter-annual climate variability. Both fluctuations hamper the possibility of making a direct comparison. In fact, even in the absence of disturbances (e.g. fires, pests), a difference in NDVI between two observations taken before and after the intervention could be due to the intervention itself, the stage of development of the vegetation at those particular times of observation, and the weather conditions experienced by the vegetation in the weeks/months preceding the observations. Assuming that climatic conditions are rather homogeneous in the neighbourhood of the restoration project, the problem can be approached by comparing the conditions of the restoration area before and after the intervention with those of similar areas nearby, as in Zucca et al. (2015). The rationale is that the anthropogenic intervention will cause a different pattern of change from before to after the intervention compared with natural changes in undisturbed and similar areas. This concept forms the basis of the before/after control/impact (BACI) sampling design (Underwood, 1992), originally developed in ecology to assess the impact of a stress (typically induced by industrial activities) on the environment. BACI has been successfully applied to statistically evaluate potential environmental and ecological impacts (Smith, 2002), but has not been used by the RS community so far.

In this study we make use of the BACI design to develop a method to assess the impact of a restoration intervention on vegetation fractional cover solely based on RS information (i.e. NDVI). The method is intended to perform a cost-effective verification of the effectiveness of the restoration intervention that may be used as a first screening, usable to plan additional field verification campaigns, and as a medium- to long-term impact monitoring tool when applied repeatedly over time. It is acknowledged that the proposed method is suited to restoration interventions that involve an increase in vegetation cover, which is not the case for a number of intervention types (e.g. a green landscape of invasive species where the restoration would aim to change the plant community composition; soil conservation measures such as rock dams to stop gully erosion).

To illustrate the approach we apply it to a case study in Senegal, where a number of restoration interventions were performed in the context of the Great Green Wall for the Sahara and the Sahel Initiative (GGWSSI), a pan-African initiative to combat desertification (African Union & Pan-African Agency of the Great Green Wall, 2012). The biophysical impact was assessed using RS data at two different spatial resolutions, namely the Moderate Resolution Imaging Spectroradiometer (MODIS) at 250 m and Landsat at 30 m, and compared with qualitative information from field observations and photointerpretation of VHR imagery. The pros and cons of using MODIS and Landsat data are discussed.

## 2 Study area

**Fig. 1:**
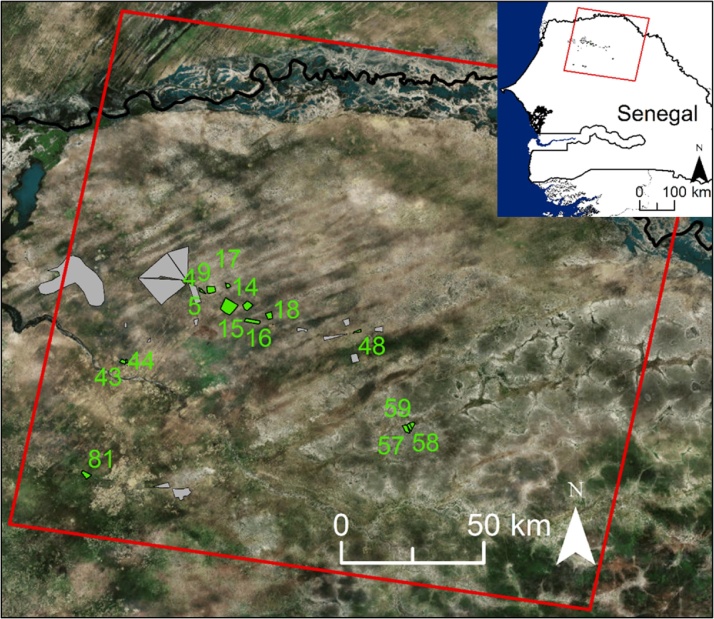
Location of the interventions considered in the case study (green polygons and identification number, details in Section 3.2). Areas with insufficient documentation about the timing of the intervention, interventions smaller than 0.25 km^2^, and areas subjected to other intervention types (i.e. conservation) are in grey. The red box delineates the boundaries of the Landsat imagery used. Background imagery is a true colour composite (source: Esri). (For interpretation of the references to colour in this figure legend, the reader is referred to the web version of this article.)

The test case-study encompasses several interventions conducted in the Linguère department of the Louga region and in the Ranerou department of the Matam region of northern Senegal (Fig. 1). The relatively flat study area belongs to the Sahelian acacia savannah ecoregion (Olson et al., 2001), and is characterised by a hot arid desert climate (BWh) according to the updated Köppen–Geiger climate classification (Peel et al., 2007). Mean annual temperature and precipitation in the study area range from 27 to 28 °C (ECMWF ERA-Interim over the period 1990–2014; Dee et al., 2011) and from 270 to 390 mm (CHIRPS rainfall estimates over the same period; Funk et al., 2015). The majority of precipitation falls during the rainy season, which occurs between July and September, and is related to the West African Monsoon (Nicholson, 2013). In the area, several restoration projects, including reforestation and improved forage production, have been implemented between 2007 and 2011 in the context of the GGWSSI by the Great Green Wall agency under the responsibility of the Senegalese Ministry of Environment. However, the technical rationale for the selection of projects and the complete description of the projects (where, what, how, success rate, etc.) is, to our knowledge, not available.

## 3 Data

### 3.1 Remote sensing

The analysis was performed on freely available satellite imagery at two different spatial scales: 250-m MODIS NDVI product and 30-m surface reflectances from the Landsat missions. For the moderate resolution, we used the eMODIS product provided by the United States Geological Survey (USGS) and based on MODIS data acquired by the Terra satellite. The product is a 10-day maximum value NDVI composite (Jenkerson et al., 2010) temporally smoothed with the Swets algorithm (Swets et al., 1999). Composites are produced every five days, resulting in six temporally overlapping composites per month. Here we only used the composites for days 1–10, 11–20, and 21-last day of each month. Both the time series of 10-day observations and the maximum annual NDVI value, representing vegetation peak development, were used in the analysis.

Inspection of MODIS multi-annual temporal profiles for the intervention areas permitted us to determine the period of vegetation growth, which roughly ranges from June to September, with maximum development reached in late August. Cloud-free Landsat imagery was selected during this period. Landsat 8 Operational Land Images (OLI) data are available since 2013; before then we had to rely on Landsat 5 Thematic Mapper (TM) and Landsat 7 Enhanced Thematic Mapper Plus (ETM+) data. However, Landsat 7 ETM+ imagery collected after 31/05/2002 has data gaps due to the Scan Line Corrector failure (SLC-off; Andrefouet et al., 2003). The issue does not prevent the analysis but has to be properly treated, as explained in the methods section.

Although not strictly required, the BACI design benefits from having multiple time observations before and after the time of intervention. Whereas gathering multiple MODIS observations is straightforward, it is very challenging for Landsat 5 and 7 in these geographical settings where the availability of cloud-free images during the growing season is very limited. For instance, a large data gap exists between 2003 and 2007 and between 2007 and 2012, when not a single cloud-free image is available in the period of maximum vegetation development. The list of the Landsat images used in the analysis is presented in Table 1.

**Table 1:** Acquisition date and sensor of Landsat data used (path 204 and row 49).

Sensor	Date
Landsat 7 ETM+	19/07/2003
Landsat 7 ETM+	16/09/2007
Landsat 7 ETM+	13/09/2012
Landsat 8 OLI	24/09/2013
Landsat 8 OLI	11/09/2014

Landsat-based NDVI was computed using the red and near infrared bands of surface reflectance products (USGS, 2016a, USGS, 2016b) retrieved from the United States Geological Survey. Largely cloud-free imagery was selected, and the CFmask band of the surface reflectance product was used to mask sparse clouds and cloud shadows.

In summary, MODIS and Landsat-based analyses differ in three aspects: *i)* the spatial resolution (250 m vs. 30 m), *ii)* the RS variable used (maximum seasonal NDVI vs. NDVI at a specific, and data availability-driven, date during the season), and *iii)*, the temporal period covered before and after the intervention (up to five years of acquisitions vs. a single acquisition).

Finally, to check the consistency of BACI results, VHR imagery from Google Earth (GE) was used for the qualitative and visual evaluation of the restoration interventions. The visual analysis of VHR imagery before and after the intervention date aimed to spot signs of interventions, ranging from signs of tractor ploughing to visible patterns of regular plantations and the growth of new trees. When imagery before the intervention was not available in GE (8 cases out of 15), the assessment of the intervention was performed on the imagery only after the intervention, and was based on a comparison of the vegetation cover inside the intervention area with that of the area outside, with obvious limitations on the possible interpretation.

### 3.2 Field missions and analysed interventions

The outline of the project polygons and the main project information (type and year of intervention) were obtained during three field visits (2014–2015) performed by the French National Centre for Scientific Research (CNRS) and supported by the Senegal State Service of Water and Forests. As a centralised and public record of restoration projects does not exist, the location of the intervention projects to be visited was defined with the staff of the Senegal State Service of Water and Forests and the Senegalese National Great Green Wall Agency. This preliminary information was complemented by visual interpretation using VHR satellite imagery from GE before the field campaigns and interviews with local communities during the campaigns. Project areas were then delineated in the field using GPS.

Restoration interventions mainly involved tree plantations (*Acacia nilotica*, *Acacia senegal*, *Acacia seyal* and *Balanites aegyptiaca*), the fencing of plots to enhance the natural regeneration of woody species and restore rangeland grasses, and the combination of the two. Tree planting usually occurred in August, during the rainy season. Activities were designed to improve land productivity over the long run under the hypothesis that the increase in vegetation cover due to the intervention would restore soil fertility and at the same time provide relevant ecosystem services for local communities (e.g. gum arabic production from *Acacia Senegal*, fruits from *Balanites aegyptiaca*, and grass straw to be harvested at the end of the season and either used or sold). Restoration interventions were implemented by the Senegalese National Great Green Wall Agency within the framework of a cash-for-work programme. It is noted that one of the interventions considered in the test case (i.e. project no. 81 of Fig. 1) does not belong to the GGWSSI, but refers to an *Acacia Senegal* plantation implemented by a private company for the production of gum arabic.

To test the proposed methodology, from the list of identified intervention sites we selected those with the following characteristics: *i)* having documentation of the period of intervention, *ii)* covered by Landsat path 204 and row 49, *iii)* implemented after year 2002, and *iv)* with an area greater than 0.25 km^2^ (i.e. a minimum of four MODIS pixels). This resulted in a total of 15 interventions (green polygons in Fig. 1). Interventions without such characteristics and an area subjected to natural conservation (grey polygons in Fig. A) were excluded from the control site search algorithm described in the methods section. A brief description of the various projects, including the time and type of intervention, field mission and VHR analysis evaluation, is presented in the results section (Table 3).

A qualitative evaluation of the success of the intervention is available for five sites that were visited in October 2015 and August 2016. Various elements were taken into account in this evaluation: presence and health status of newly planted trees, tree and herbaceous cover difference with respect to surroundings, informal interviews with locals. This information, together with the visual interpretation of VHR imagery, was used to carry out a consistency check with the results of the proposed methodology.

## 4 Methods

In BACI design, to account for natural changes, the NDVI of the restoration intervention area (i.e. the “impact” site) is compared to another site, which is referred to as the “control” site (Smith, 2002). The use of multiple control sites (i.e. BACI with multiple sites) extends this idea and avoids the criticism that the results of the BACI experiment are solely due to a poor choice of the control site. The location of controls is selected randomly among sites that are similar to the impact site (details in Section 4.1).

### 4.1 Spatial sampling

With respect to the impact site, a control area should have the following characteristics:

i) similar land cover before the intervention;
ii) relatively close in space in order to experience the same weather variability;
iii) not subjected to anthropogenic changes during the whole before–after period being analysed;
iv) randomly selected.

In addition, even if not strictly required by the BACI design, we opted for selecting control areas with a size similar to that of the impact area to ensure a more balanced sampling size. Similarity in soil characteristics, known to be important determinants of vegetation in arid systems, is expected to be implicitly ensured by condition *i*.

**Fig. 2:**
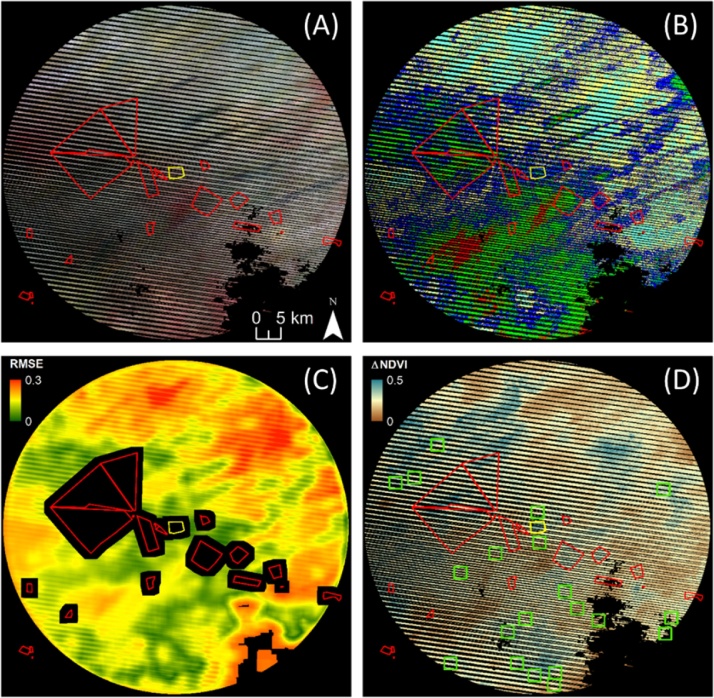
Example of intermediate results of the described processing for project no. 9 (yellow polygon, other projects in red). Landsat images are from the following dates: 19/07/2003 (before), 13/09/2012 (after). (A) near infrared false colour composition Landsat image before; masked pixels (i.e. outside search area, SLC-off, detected as clouds or cloud shadows in the before or after imagery) are in black, "stripes” are originated by SLC-off affected pixels; (B) five classes ISODATA classification of the valid pixels; (C) class composition RMSE with respect to the intervention area; RMSE of the window assigned to the central pixel; pixels whose window would overlap other projects are masked out (black); (D) green square polygons are the selected controls; NDVI difference (value after–before) in the background. (For interpretation of the references to colour in this figure legend, the reader is referred to the web version of this article.)

In order to fulfil these requirements, we proceed as follows for each of the impact sites. When different settings are used for the MODIS and Landsat analysis, this is explicitly mentioned in the text and reported in Table 2. Some of the intermediate products of the analysis for the Landsat data and impact site number 9 are shown in Fig. 2 as an example.

**Table 2:** List of MODIS- and Landsat-specific parameters used in the analysis.

	MODIS	Landsat
Ratio *r* between search and impact area	600	
Number *n* of ISODATA classes	5	
Similarity threshold *s*	0.9	
Number of controls randomly extracted (*n*_*c*_)	20	
Target variable	Maximum annual value of smoothed NDVI	NDVI of within-season imagery
Additional cloud screening	None	Visual analysis
Temporal sampling (before and after)	5 + 5 samples	1 + 1 sample

First, we restrict the area from which controls are selected to a circular area centred on the centroid of the impact site. Pixels affected by cloud contamination and SLC-off in either the before or after imagery are masked out (Fig. 2A). The restriction to such a circular area has the objective of fulfilling condition *ii*, i.e. defining “a neighbourhood” where climatic conditions should not significantly change. The extent of this area is defined as a multiple of the impact area size (search area/impact area = *r*). We made the search area proportional to the size of the impact area to ensure that it contains a roughly constant number of potential controls, independent of the impact area size. The ratio *r* was set for this study to 600. If the impact area had a circular shape, this would correspond to a ratio between the area searched and the impact radius of 24.5. In the case study, this resulted in an average search radius of 25 km (range = 9–61 km), where similar climatic conditions can be reasonably expected.

Second, we use the images acquired in the period before the intervention (Fig. 2A) to perform an iterative self-organised unsupervised clustering algorithm (ISODATA) with *n* classes spatially restricted to the search area (Fig. 2B). With a trial and error process based on the qualitative comparison of the ISODATA classification map and VHR imagery, we set *n* = 5 in this study. A larger number of classes can be selected if the landscape is more heterogeneous. To fully compare the results gathered with the Landsat and the MODIS analysis, we perform the classification using either Landsat or MODIS data for the two types of analysis, implying that different control sites are selected. For the Landsat analysis, all the bands in the reflected domain of a single image are used for the classification, whereas for MODIS we follow the approach proposed by de Bie et al. (2011), using the multi-temporal NDVI trajectory instead of the multispectral information. The classification is thus performed on a five-year multi-temporal dataset of 10-day composites ending the year before the implementation of the restoration action. After this classification stage, the fractional class composition of the impact area is computed.

Third, we define a generic control as a square spatial window with the same area of the impact site. The population of potential controls is thus formed by all the possible and overlapping windows centred on each of the pixels belonging to the search areas. Potential controls that overlap other impact sites or excluded areas (the area subject to environmental conservation in our case study) are excluded. Potential controls having more than 50% of invalid pixels (as they are covered by the cloud and shadow mask) are also excluded. Then, the fractional class composition is computed for each potential control.

Fourth, the land cover similarity between each potential control and the impact is defined as the complement of the root mean square error between the fractional compositions and one, i.e. similarity *s* = 1–RMSE (Fig. 2C). Values close to one thus indicate nearly identical overall class composition of a potential control and the impact. Note that the similarity of NDVI values before the intervention is not considered here as the BACI design does not require similar levels of the variable of interest.

Fifth, we subsample the population of potential controls by discarding those with a similarity smaller than *s* (0.9). At this point we have a sample of potential controls that fulfil conditions *i* and *ii*. From this sample we randomly extract *n*_*c*_ control sites (*n*_*c*_ = 20 in this study, Fig. 2D). Random extraction is executed using probability proportional to size sampling (Lohr, 2010), in which the selection probability for each element is proportional to its similarity to the impact site. In this way, the most similar controls have a higher probability of being selected. Once a control is extracted, all its overlapping potential controls are excluded for further selection and the random extraction is repeated until all the required controls are selected. It is noted that this procedure does not guarantee that all desired *n*_*c*_ controls are actually available. If the number of selected controls is considered to be insufficient, one may increase the search area or reduce the required similarity *s* to increase the population of candidate controls and thus the number of selected controls.

Once the location of the controls is established, the NDVI is extracted for all valid pixels belonging to the impact and control areas for the period before and after the intervention. The selection process described so far was implemented in IDL (Harris Geospatial Solution, Inc.) and fully automatised.

Finally, condition *iii* was tested by visually inspecting the available time series of Landsat imagery of the selected control sites. It is noted that only clear land use changes, for instance from natural vegetation to cropland or to settlements, are detectable in such a way. The possible occurrence of less visible changes, such as unreported rangeland management practices, can therefore not be excluded. The impact of the potential selection of such unsuitable controls is expected to be mitigated by gathering a relatively large number of control sites.

### 4.2 Treatment of Landsat 7 SLC gaps

An estimated 22% of the Landsat 7 scenes is lost because of the SLC failure (http://landsat.usgs.gov/products_slcoffbackground.php). The SLC-off effects are most pronounced along the edges of the scene and gradually diminish toward its centre. The precise location of the missing scan lines varies from scene to scene. Therefore, it is difficult to anticipate the fraction of missing data for individual impact areas. With our test cases, the fraction of missing data varied between 0% and 40% and operated as a random subsampling with no expected consequences on the following BACI test. In the presence of this SLC problem, the affected pixels were considered as belonging to an additional land cover class, thus contributing to the similarity measure described above. In this way we favoured the selection of controls showing a similar fraction of SLC-affected pixels.

### 4.3 Temporal sampling

Multiple temporal sampling before and after the putative impact is preferable as it ensures that coincidental temporal fluctuations in either location do not confound the detection of the impact (Underwood, 1992). Due to the limited frequency of temporal acquisition, we could not retrieve multiple observation imagery before and after the intervention from Landsat, and we consequently applied BACI based on a single couple of before–after observations and multiple control sites. The closest cloud-free images before and after the time of intervention were thus selected for each restoration site. The more robust BACI design, with observations from multiple dates and sites, was instead used with the high temporal frequency MODIS data. That is, up to five annual values of maximum annual NDVI were extracted from the MODIS time series.

### 4.4 Statistical analysis

A linear mixed-effects model on NDVI site-level averages was used to test the impact of the restoration intervention as in Schwarz (2015). In this context, the period (before/after), the site class (impact/control) and the interaction of site class and period are fixed effects while the site and the sampling time, being non-exhaustive samples of the potential sites and sampling times, are considered to be random effects. Linear mixed-effects models use maximum likelihood to estimate the parameters of the linear function containing both fixed and random effects. Output is in the form of approximate z-ratios or normal deviates, which allows statistical tests on any linear combination of the fixed parameters (Pinheiro and Bates, 2000). To evaluate the impact of the intervention we were interested in the interaction of the period and the site class (the so-called BACI effect) representing the differential change between impact and control sites compared before and after the intervention. The (null) hypothesis of no change was rejected at the conventional 5% significance level.

The BACI analysis provides two important statistics (among others): the significance level (i.e. *P*-value) of the BACI effect test (i.e. no change null hypothesis) and the BACI contrast. The BACI contrast is calculated as the difference (controls vs. impact) between the mean differences (after vs. before):

(1) $$\text{BACI}\;\text{contrast}=(\mu_{\text{CA}}-\mu_{\text{CB}})-(\mu_{\text{IA}}-\mu_{\text{IB}})$$

Where μ is the site-specific spatial mean of the variable selected to represent the impact (here NDVI); CA, IA stand for Control and Impact After, respectively; CB and IB for Control and Impact Before, respectively. By convention, a negative contrast indicates that the variable has increased more (or decreased less) in the impacted site with respect to controls in the time period ranging from before to after the implementation of the restoration project. The BACI contrast is expressed in the same units of the variable of interest, here NDVI. In order to highlight the magnitude of the contrast with respect to the initial conditions, we normalise it by the mean of the NDVI of the impact area before the intervention took place (μ_IB_) and express it as a percentage. This derived variable is referred to as “relative contrast” in the following.

It is noted that, despite the fact that NDVI computed from Landsat 7 and 8 (ETM+ and OLI sensors) may be slightly different because of the different spectral responses of the bands (Roy et al., 2015) and different atmospheric correction algorithm, this impacts both the project site and the controls and hence does not have an effect on the BACI analysis, which works on the difference between the two types of area.

The open source statistical software R (R Development Core Team, 2016) was used to develop a script to automatise the statistical test following Schwarz (2015).

## 5 Results and discussion

Results of the BACI analysis, along with project information, VHR photointerpretation and field mission qualitative evaluation, are reported in Table 3. The number of control sites excluded from the analysis after visual inspection ranged from zero to a maximum of six. The anthropogenic changes detected in the period after the intervention mainly refer to appearance of agricultural fields and settlements.

**Table 3:** Main information of analysed interventions, field mission evaluation, visual interpretation of Google Earth VHR imagery and BACI results on MODIS and Landsat data. n.a. stands for not available. The mean of the RS variable is computed as the overall mean extracted before the intervention (all sites, all sampling dates). Green (likely success), light green (moderate or ambiguous success) and grey background (likely failure) is used to rank the intervention's success based on the field mission and VHR qualitative evaluation. Green background is used in the BACI section to highlight negative BACI contrasts (in bold) that are significant at the 0.05 *P*-value. Grey background indicates a non-significant BACI effect.

### 5.1 BACI analysis

A significantly negative BACI contrast (i.e. improvement in NDVI with respect to controls after the intervention) was detected in five and four out of 15 sites using MODIS and Landsat data, respectively. For the majority of sites, the (null) hypothesis of no change could not be rejected. For three sites, the contrast was indeed positive, i.e. there was a relative decrease in NDVI in the restoration area.

Focussing on the sites for which a significant BACI effect was detected, the average relative contrast is −20% and −27% for MODIS and Landsat data, respectively. Considering NDVI as a rough approximation of the fractional vegetation cover, these numbers translate into a significant improvement in the vegetation cover with respect to the controls.

**Fig. 3:**
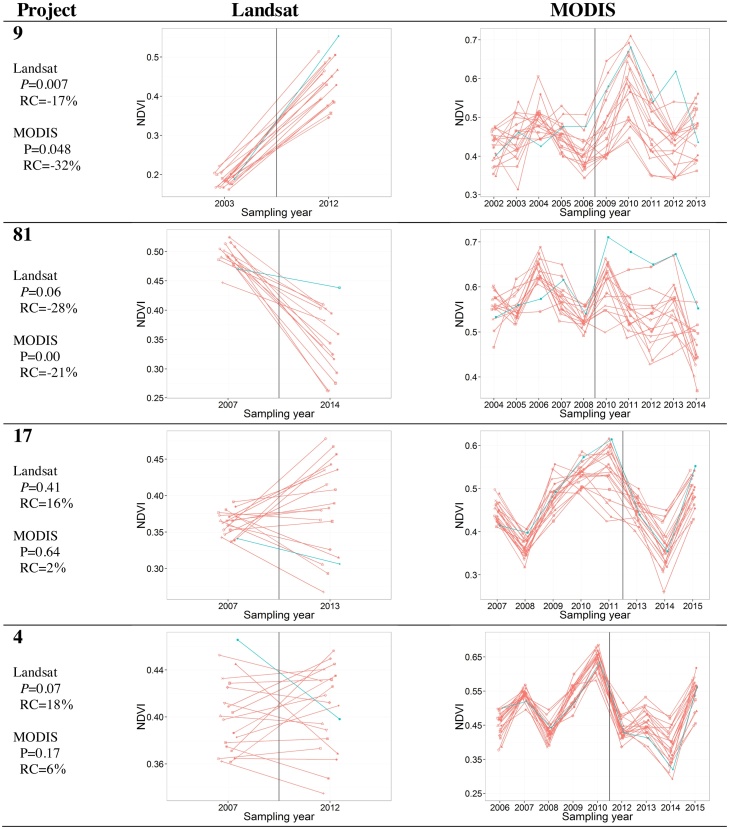
Temporal profiles of mean NDVI values for selected impact (blue lines) and corresponding control sites (red lines) for Landsat (left) and MODIS (right) data. Sampling dates before and after the intervention are separated by the vertical black line. The *P*-value (*P*) and the percent relative contrast (RC) are reported. (For interpretation of the references to colour in this figure legend, the reader is referred to the web version of this article.)

As an example of the data used for the BACI analysis, impact and control averages are shown in Fig. 3 for four representative interventions: no. 9, where a significantly negative BACI effect is found using both Landsat and MODIS; no.81, where the negative contrast is significant at the 0.05 level for MODIS only (*P* < 0.1 for Landsat); no. 17 with a positive but non-significant contrast; and finally no. 4 with a positive and non-significant contrast (*P* < 0.1 for Landsat).

In order to gain insights into the difference between the MODIS and Landsat analyses we focus on the agreement between two relevant BACI statistics (i.e. contrast and *P*-value). First, Table 3 shows a perfect match in the BACI contrast sign. That is, both types of analysis agree in the evaluation of the sign of the intervention, either re-greening (negative contrast) or degradation (positive contrast) of the impact site compared to the controls. The magnitude of the contrast and the mean of the RS variable can be different between the two types of analysis because the RS variable is different: the maximum seasonal NDVI for MODIS, and the NDVI value during the growing season at a specific sampling date dictated by image availability for Landsat.

Second, large agreement in the detection of a significant re-greening of the intervention (i.e. negative BACI contrast with *P*-value < 0.05) exists between the two types of analysis. Only one case of minor disagreement is found for site no. 81 (Table 3 and Fig. 3), for which the visual analysis of Google Earth VHR imagery indicates that the plantation was actually implemented. In this case both types of analysis compute a nearly identical and negative BACI contrast (−0.12) whereas they differ in the significance level attributed. However, the *P*-value of Landsat (0.058) is not far from the threshold (0.05) used to reject the (null) hypothesis of no change. As a result, the change detected using Landsat data has a lower confidence level (*P* < 0.10).

**Fig. 4:**
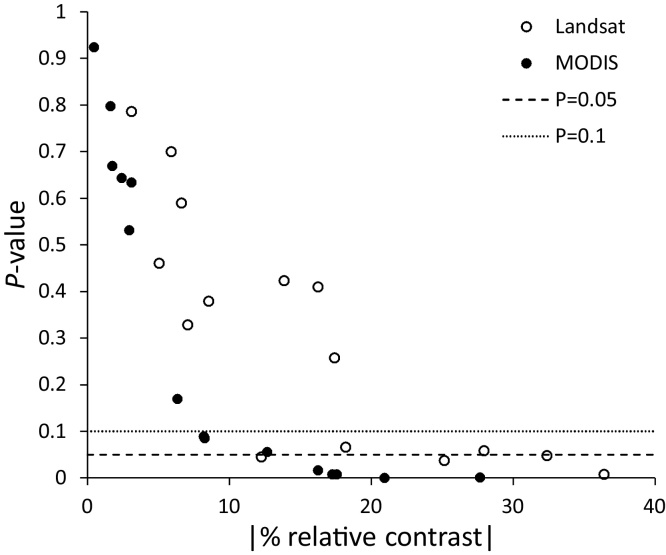
Scatterplot of the absolute value of the relative BACI contrast (equal to 100 * |contrast|/mean of RS variable before the intervention) vs *.* the *P*-value of the BACI test. Null hypothesis rejection of typical *P*-value thresholds of 0.05 and 0.1 are shown as dashed lines.

Other minor differences (i.e. not leading to different test outcome) between the results of the two types of analysis refer to different magnitudes of the *P*-value. The *P*-value of MODIS is generally lower than that of Landsat. Fig. 4 shows the *P*-value of the two types of analysis vs *.* the absolute value of the relative BACI contrast.

Both types of analysis show a reduction in the *P*-value with increasing absolute value of the relative contrast, as the test essentially builds (also) on the magnitude of the BACI contrast. However, the MODIS *P*-values are mostly lower than those of Landsat for similar relative contrasts. Therefore, the multiple temporal sampling that can be achieved using MODIS data appears to be instrumental in increasing the significance level of the test with respect to the single time analysis of Landsat. This is likely due to three reasons: *i)* increased sample size for MODIS analysis, *ii)* better representation of the overall vegetation cover offered by the maximum NDVI with respect to the single date NDVI, and *iii)* reduced dependency of MODIS on a specific year and time. Concerning the latter, with the MODIS set-up we analyse the control/impact differential behaviour in a multi-year time span, making it less sensitive to possible year-specific peculiarities that may affect the single-year high-resolution Landsat set-up (see Fig. 3). It is noted that the same multiple-period design can be applied to high-resolution freely available data in geographical settings with a higher availability of cloud-free Landsat imagery, or when analysing more recent projects that can exploit the more frequent availability of Landsat 8 imagery and other recently available instruments (e.g. Sentinel 2). The uncertainties connected to the use of a single image before and after the intervention are well exemplified by the temporal evolution of NDVI for project no. 81 in Fig. 3. A non-significant BACI effect is detected using the single-image set-up of Landsat (*P* = 0.058) despite a quite large negative relative contrast (−27.95%). For the same site, the MODIS multi-year profile shows a large inter-annual variability. The Landsat single-image set-up picked up 2007 as the year “before”, when the control had the third-highest MODIS NDVI. If a different year were available, for example 2006 when the control had the second-lowest MODIS NDVI, this may have resulted in a different (lower) *P*-value.

Project no. 4 (showing degradation with respect to controls) shows an opposite behaviour: lower *P*-value (i.e. higher confidence) for the Landsat analysis. Here, the small size of the restoration area plays a role, resulting in a poor MODIS spatial sampling, on the one hand by reducing the sample size and the power of the test, and on the other by making the few MODIS samples less reliable. In fact, the actual area sensed by the instrument is greater than the nominal spatial resolution, and has an elliptical shape controlled by the sensor characteristics and observation geometry (Duveiller et al., 2011, Duveiller and Defourny, 2010). Thus, a fraction of the signal in pixels located at the border of the project area may originate from an area outside. This effect may be non-negligible when the project area is composed of only a few MODIS pixels, as for project no. 4.

Besides the statistical test result (i.e. rejection of the null hypothesis of no change), the relative BACI contrast can provide additional insights into the extent of the success of a given intervention project. For instance, with MODIS analysis, this ranges from +6.3% (degradation for project no. 4) to −27.7% (improvement for project no. 14), indicating a different magnitude of the effect of the different restoration interventions.

### 5.2 BACI results vs. qualitative information

A general agreement between the qualitative information extracted from Google Earth VHR imagery and BACI results is observed. In all sites where no signs of interventions or no difference with the surrounding areas was observed in VHR imagery, the BACI effect is not significant. In all sites where a pattern of regularly planted and established trees were observed, the BACI contrast is negative and the BACI effect is significant, with the exception of site no. 81 which is not significant when Landsat is used. The test of the BACI effect also agrees with the field qualitative evaluation available for five sites. Among the five sites, two were evaluated as being relatively successful and are matched by a significant BACI effect (sites no. 14 and 15), and three were negatively evaluated and are matched by a non-significant BACI effect (sites no. 9, 16 and 44). Site no. 5, where the presence of reforestation intervention was not visible, was instead found to have a significant and negative BACI. However, the field evaluation did not provide any information about the grassland cover that may have improved after the fencing intervention, thus triggering the statistical detection of a greening effect.

### 5.3 Applicability of the method to different intervention types

Albeit restoration interventions that do not involve a “greening” cannot be scrutinised using NDVI, the range of applicability may be expanded using the same statistical framework with other RS-based quantitative indicators, when considered relevant for assessing the success of a specific type of intervention at a given scale of analysis. For instance, soil erosion processes could be assessed by detecting erosion features and eroded areas or by estimating erosion-controlling factors, such as soil moisture and surface roughness (Anderson and Croft, 2009, Vrieling, 2006). Spatial pattern metrics could support the assessment of restoration interventions that impact habitat composition, fragmentation, and connectivity at landscape level, also in relation to land degradation processes (Fava et al., 2015, Kéfi et al., 2007). As an additional example, fine-scale quantitative mapping of specific plant species (e.g. invasive) could be critical to monitor the effectiveness of plant removal or control efforts (Pysek and Richardson, 2010).

### 5.4 Applicability of the method to different landscape settings

Topographic variations are not explicitly accounted for in the described method. Although not an issue in the flat case-study landscape, two effects of topography can be envisaged in regions with significant relief. First, different vegetation types grow in locations with different elevation, slope and aspect. Thus, controls should be selected with similar topographic characteristics with respect to the restoration site. As we expect different vegetation types to be spotted by the classification of RS imagery, this first effect does not hamper the proposed method. In addition, in the cases where such topographic characteristics are expected to be important, they could be added to the input layers of the classification. The second effect of topography is on the geometry of the sun-target-sensor system, and thus on the reflectance. Moderate relief variations are expected to have a minor impact on the method as the use of a band ratio such as the NDVI will reduce the topographic effect (Lee and Kaufman, 1986). In addition, different illumination conditions (at least those related to the direct light component) can be normalised using, for instance, slope-aspect corrections (e.g. Teillet et al., 1982). Thus, topography can be treated and does not limit the applicability of the method.

The BACI analysis is a comparative method in which the temporal variability due to natural environmental conditions (i.e. weather) is accounted for using controls. The random selection of multiple control sites and the visual inspection of their stability over the analysis period minimises the impact of the selection of unsuitable controls (i.e. affected by non weather-driven changes in greenness after the time of intervention). However, if the landscape around the restoration area is subjected to widespread anthropogenic changes (e.g. agricultural intensification, urbanisation), the possibility of selecting multiple suitable controls will be severely limited, affecting the discrimination power of the test. On the contrary, possible natural disturbances such as fires or pests can be accounted for by the test. In fact, a decrease in greenness would be detected if the disturbance affected only the restoration site while a relative increase would be more likely to be detected if the disturbance affected several controls. The change in greenness may be then interpreted as decreased (or increased) vulnerability to such disturbances due to the intervention.

## 6 Conclusions

For the first time, a before/after control/impact (BACI) design was applied to RS data to evaluate the biophysical impact of restoration projects. Large agreement was found in the statistical test outcomes using either MODIS or Landsat data. The availability of frequent MODIS observations makes the data of this instrument well suited to the most robust BACI design, exploiting multiple controls and multiple observations before and after the intervention. The use of Landsat data in our test case study was limited by the poor availability of cloud-free imagery, compelling the application of a single-time BACI design and resulting in generally lower confidence (i.e. higher significance level, *P*-value) of the test results. The analysis of more recent intervention projects will benefit of the availability of more frequent satellite observations from Landsat 8 and Sentinel 2 satellites. The combination of high spatial and temporal resolution offered by sensors such as the Sentinels 2 may considerably increase the potential of the proposed method. In addition, for earlier project, the use of commercial satellite (e.g. SPOT 4 and 5, Rapid Eye) may be considered to complement the free imagery and increase data availability.

Results of the statistical analysis were in agreement with the qualitative information provided by field observations and visual interpretation of the VHR imagery in Google Earth. The proposed approach can be considered a first screening of restoration interventions that may drive further and complementary in situ analyses, thus increasing the cost-efficiency and feasibility of the evaluation of restoration interventions. In addition, the methodology can be used for the long-term monitoring of restoration interventions, thus allowing the benefits of the initial investment and its sustainability to be evaluated.

When NDVI is used, the applicability of the proposed method is limited to the verification of a biophysical impact in terms of variation in vegetation cover. This is not limited to reforestation and rangeland improvement but to a range of interventions (e.g. soil conservation, surface water run-off control, infrastructures for irrigation, improved land governance and management, etc.) that also cause re-greening. The use of other remote-sensing-derived variables (e.g. soil moisture, surface roughness, fragmentation, VHR plant species mapping) may further extend the applicability of the statistical framework to other aspects of restoration interventions. In situ analyses remain of fundamental importance, not only to provide a more detailed set of biophysical indicators targeted at the specific restoration, but also to consider other key aspects of restoration related to social perception and economic impacts.
